# Diffuse paediatric-type high-grade glioma, H3-wildtype and IDH-wildtype: case series of a new entity

**DOI:** 10.1007/s10014-023-00468-3

**Published:** 2023-08-10

**Authors:** Katja Bender, Johannes Kahn, Eilís Perez, Felix Ehret, Siyer Roohani, David Capper, Simone Schmid, David Kaul

**Affiliations:** 1 Department of Radiation Oncology, Charité-Universitätsmedizin Berlin, Corporate Member of Freie Universität Berlin and Humboldt-Universität zu Berlin, Charitéplatz 1, 10117 Berlin, Germany; 2 Department of Radiology, Charité-Universitätsmedizin Berlin, Corporate Member of Freie Universität Berlin and Humboldt-Universität zu Berlin, Charitéplatz 1, 10117 Berlin, Germany; 3 Department of Neuropathology, Charité-Universitätsmedizin Berlin, Corporate Member of Freie Universität Berlin and Humboldt-Universität zu Berlin, Charitéplatz 1, 10117 Berlin, Germany; 4 Berlin Institute of Health, Charité-Universitätsmedizin Berlin, Charitéplatz 1, 10117 Berlin, Germany; 5 German Cancer Consortium (DKTK), Partner Site Berlin, German Cancer Research Center (DKFZ), Heidelberg, Germany

**Keywords:** Diffuse paediatric-type high-grade glioma, H3-wildtype, IDH-wildtype, pHGG, Case series

## Abstract

Diffuse paediatric-type high-grade glioma, H3-wildtype and IDH-wildtype (pHGG) is a rare and aggressive brain tumor characterized by a specific DNA methylation profile. It was recently introduced in the 5th World Health Organization classification of central nervous system tumors of 2021. Clinical data on this tumor is scarce. This is a case series, which presents the first clinical experience with this entity. We compiled a retrospective case series on pHGG patients treated between 2015 and 2022 at our institution. Data collected include patients’ clinical course, surgical procedure, histopathology, genome-wide DNA methylation analysis, imaging and adjuvant therapy. Eight pHGG were identified, ranging in age from 8 to 71 years. On MRI tumors presented with an unspecific intensity profile, T1w hypo- to isointense and T2w hyperintense, with inhomogeneous contrast enhancement, often with rim enhancement. Three patients died of the disease, with overall survival of 19, 28 and 30 months. Four patients were alive at the time of the last follow-up, 4, 5, 6 and 79 months after the initial surgery. One patient was lost to follow-up. Findings indicate that pHGG prevalence might be underestimated in the elderly population.

## Introduction

Diffuse paediatric-type high-grade glioma, H3-wildtype and IDH-wildtype (pHGG) is a new brain tumor entity defined by a specific DNA methylation profile. It has recently been introduced in the WHO classification of central nervous system (CNS) tumors of 2021 [1].

PHGG is considered a highly malignant brain tumor (WHO grade 4), mostly occurring in children and adolescents. Histologically it is characterized as a diffusely infiltrating glioma with mitotic activity, showing no mutations in IDH1, IDH2 or H3-genes. Furthermore, the tumor either presents a methylation profile aligned with subgroups pHGG RTK1, pHGG RTK2 or pHGG MYCN or it exhibits molecular features as PDGFRA alteration, EGFR alteration or MYCN amplification [1].

Prognosis has been reported as unfavorable with a median overall survival (OS) of 17 months [2]. Subtype classification has shown pHGG RTK2 to be associated with the longest median survival time of 44 months, followed by pHGG RTK1 (median OS 21 months) and pHGG MYCN (median OS 14 months) [3].

## Materials and methods

We compiled a retrospective series of cases diagnosed between 2015 and 2022 at Charité-Universitätsmedizin Berlin. All patients classified as pHGG by DNA methylation-based brain tumor classification analysis were included [4]. Except for one case, the cases included had 12.5 classifier scores between 0.89 and 1.00. Case 1 showed a score of 0.7. In conjunction with the results of histology and copy number analysis, we decided to include this case in the series. Mutations of either IDH1, IDH2 or H3.3 were ruled out for all cases by pyrosequencing. Because pHGG has only been established as a WHO diagnosis in 2021, most tumors in our study group were not initially categorized as pHGG and only later identified as pHGG. Demographic data, information on clinical symptoms, histopathological and immunohistochemical testing, neuroradiological imaging, clinical course and outcome data were obtained from institutional medical records. Dates of death were acquired from the municipal death register. Progression-free survival (PFS) was defined as the time span between primary surgery and the first tumor recurrence on magnetic resonance imaging (MRI). OS was calculated from the day of primary surgery to the date of death.

## Results

### Patient characteristics

We identified eight patients diagnosed with pHGG between 2015 and 2022. In the same period, a total number of 1.339 patients with grade 4 glioma were diagnosed at our institution.

Table 1 provides an overview of patient characteristics.

**Table 1:** Characteristics of eight patients with diffuse paediatric-type high-grade glioma, H3-wildtype and IDH-wildtype

Patient no	Sex/age	Original neuropathological diagnosis	Methylation class	Tumor location	Surgery	KPS (%)	RT (Gy)	CTx	PFS (m)	OS (m)
1	Male/51	Glioblastoma multiforme (WHO grade 4)	pHGG, RTK1	Left central	Microsurgical resection	90	60	Concomitant TMZ + adjuvant TMZ	15	28
2	Female/8	Anaplastic astrocytoma, IDH-wildtype with molecular features of a glioblastoma, IDH-wildtype	pHGG, RTK1	Right temporal lobe + cervical spine	Stereotactic biopsy	60	45	Carboplatin + vincristine	12	Alive at last follow-up, 79 months After surgery
3	Male/45	Glioblastoma, IDH-wildtype (WHO grade 4)	pHGG; RTK2	Multifocal, right frontal lobe	microsurgical resection	80	60	Concomitant TMZ + CCNU	No progress in MRI	Alive at last follow-up, 5 months after surgery
4	Female/23	Glioblastoma, IDH-wildtype (WHO grade 4)	pHGG, MYCN	Right temporo-parietal cerebrum	Microsurgical resection	100	60	Concomitant + adjuvant TMZ	9	19
5	Male/71	Glioblastoma, IDH-wildtype (WHO grade 4)	pHGG, RTK1	Right parietal lobe	Subtotal resection	100	59.2	Concomitant TMZ + adjuvant TMZ, followed by combined TMZ-CCNU + TTF	22	30
6	Female/29	Diffuse paediatric-type high-grade glioma, H3-wildtype and IDH-wildtype (WHO grade 4)	pHGG, RTK2	Left insular lobe	Microsurgical resection	90	59.2	Concomitant TMZ + adjuvant TMZ	No progress in MRI	Alive at last follow-up, 6 months after surgery
7	Female/15	Glioblastoma, IDH-wildtype (WHO grade 4)	pHGG, MYCN	Right fronto-temporal cerebrum	Microsurgical resection	60	n.a	Adjuvant CCNU, vincristine, cyclophosphamide, adriamycin	n.a.	Lost to follow up, last seen alive: 08/19
8	Female/52	Diffuse glioma with the epigenetic profile of a diffuse paediatric-type high-grade glioma, H3-wildtype and IDH-wildtype (WHO grade 4)	pHGG, A&B	Bithalamic, right > left	Stereotactic biopsy	100	no RT	No CTx	No progress in MRI	Alive at last follow-up, 4 months after surgery
Total (*n* = 8)						Mean KPS: 85	Mean dose: 57.2			

*CCNU* lomustine, *CTx* chemotherapy, *Gy* gray, *H3* histone H3, *IDH* isocitrate dehydrogenase, *KPS* Karnofsky performance score, *m* months, *n.a.* not available, *OS* overall survival, *PFS* progression-free survival, *RT* radiotherapy mean dose, *TMZ* temozolomide

Table 2 summarizes preoperative MR-imaging features of all our cases.

**Table 2:** MRI features of eight pre-surgical pHGG-cases

Case no.	Size max. diameter in mm	MRI T2	MRI T1n	Enhancement T1 Gd	DWI	Texture		Margins	Surrounding edema	Cysts/necrosis
						T1 Gd	T2			
1	44 × 28	Hyperintense	Hypointense	Rim enhancement	No restriction	Slightly inhomogeneous	Slightly inhomogeneous	Sharp	Yes	Cystic
2	23 × 13	Hyperintense	Hyper-/isointense	Slight enhancement	n.a.	Inhomogeneous	Inhomogeneous	Diffuse	Yes	No
3	31 × 30 (largest-multifocal)	Hyperintense	Hypointense	Rim enhancement	No restriction	Homogeneous	Homogeneous	Sharp	Yes	Cystic
4	22 × 20	Hyperintense	Hypo-/isointense	Partly rim, partly solid enhancement	No restriction	Inhomogeneous	Inhomogeneous	Sharp	Yes	No
5	62 × 46	Hyperintense	Hypointense	Rim enhancement	No restriction	Homogeneous	Homogeneous	Sharp	Slight	Cystic
6	14 × 13	Hyperintense	Hypointense	No enhancement	No restriction	Slightly inhomogeneous	Slightly inhomogeneous	Diffuse	Slight	No
7	n.a. diffuse	Hyperintense	Hypo-/isointense	No enhancement	No restriction	Inhomogeneous	Inhomogeneous	Diffuse	Yes	No
8	68 × 42	Hyperintense	Hypo-/isointense	No enhancement	No restriction	Slightly inhomogeneous	Slightly inhomogeneous	Sharp	No	No

*Gd* gadolinium, *MRI* magnetic resonance imaging, *n.a.* not applicable, *n* native, *pHGG* diffuse paediatric-type high-grade glioma

**Fig. 1:**
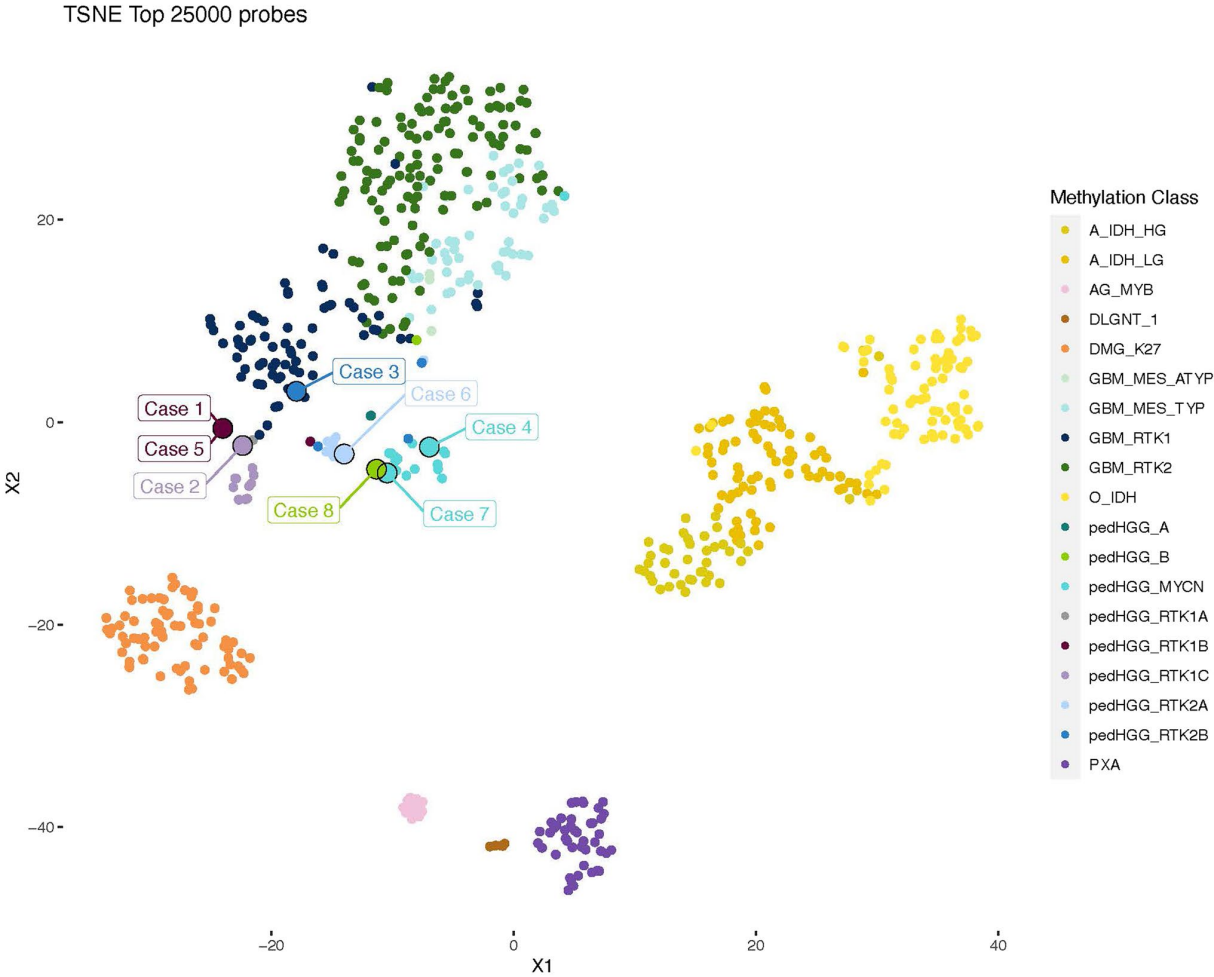
T-distributed stochastic neighbor embedding (t-SNE) analysis of DNA methylation data of the eight cases of diffuse paediatric-type high-grade glioma, H3-wildtype and IDH-wildtype in this series together with a reference cohort of 19 different molecular tumor classes (*n* = 650). Reference methylation classes: a IDH HG astrocytoma, IDH-mutant, high grade (41 cases); A IDH LG astrocytoma, IDH-mutant, low grade (81 cases); AG MYB angiocentric glioma, MYB/MYBL1-altered (14 cases); DLGNT 1 diffuse leptomeningeal glioneuronal tumor, subtype 1 (6 cases); DMG K27 diffuse midline glioma, H3 K27M mutant (74 cases); GBM MES ATYP glioblastoma, IDH-wildtype, mesenchymal subtype, subclass B (novel) (3 cases); GBM MES TYP Glioblastoma, IDH-wildtype, mesenchymal subtype (52 cases); GBM RTK1 glioblastoma, IDH-wild-type, subclass RTK1 (69 cases); GBM RTK2 glioblastoma IDH-wildtype, subclass RTK2 (134 cases); O IDH oligodendroglioma, IDH-mutant and 1p/19q-codeleted (79 cases); pedHGG A diffuse paediatric-type high grade glioma, H3-wildtype and IDH-wildtype, subtype A (2 cases); pedHGG B diffuse paediatric-type high grade glioma, H3-wildtype and IDH-wildtype, subtype B (2 cases); pedHGG MYCN diffuse paediatric-type high grade glioma, MYCN subtype (19 cases); pedHGG RTK1A diffuse paediatric-type high grade glioma, RTK1 subtype, subclass A (2 cases); pedHGG RTK1B diffuse paediatric-type high grade glioma, RTK1 subtype, subclass B (3 cases); pedHGG RTK1B Diffuse paediatric-type high grade glioma, RTK1 subtype, subclass C (11 cases); pedHGG RTK2A diffuse paediatric-type high grade glioma, RTK2 subtype, subclass A (10 cases); pedHGG RTK2B diffuse paediatric-type high grade glioma, RTK2 subtype, subclass B (4 cases); PXA (anaplastic) Pleomorphic xanthoastrocytoma (44 cases)

Figure 1 shows a t-distributed stochastic neighbor embedding (t-SNE) analysis of DNA methylation data of the eight diffuse paediatric-type high-grade glioma, H3-wildtype and IDH-wildtype together with a reference cohort of 19 different molecular tumor classes (*n* = 650).

**Fig. 2:**
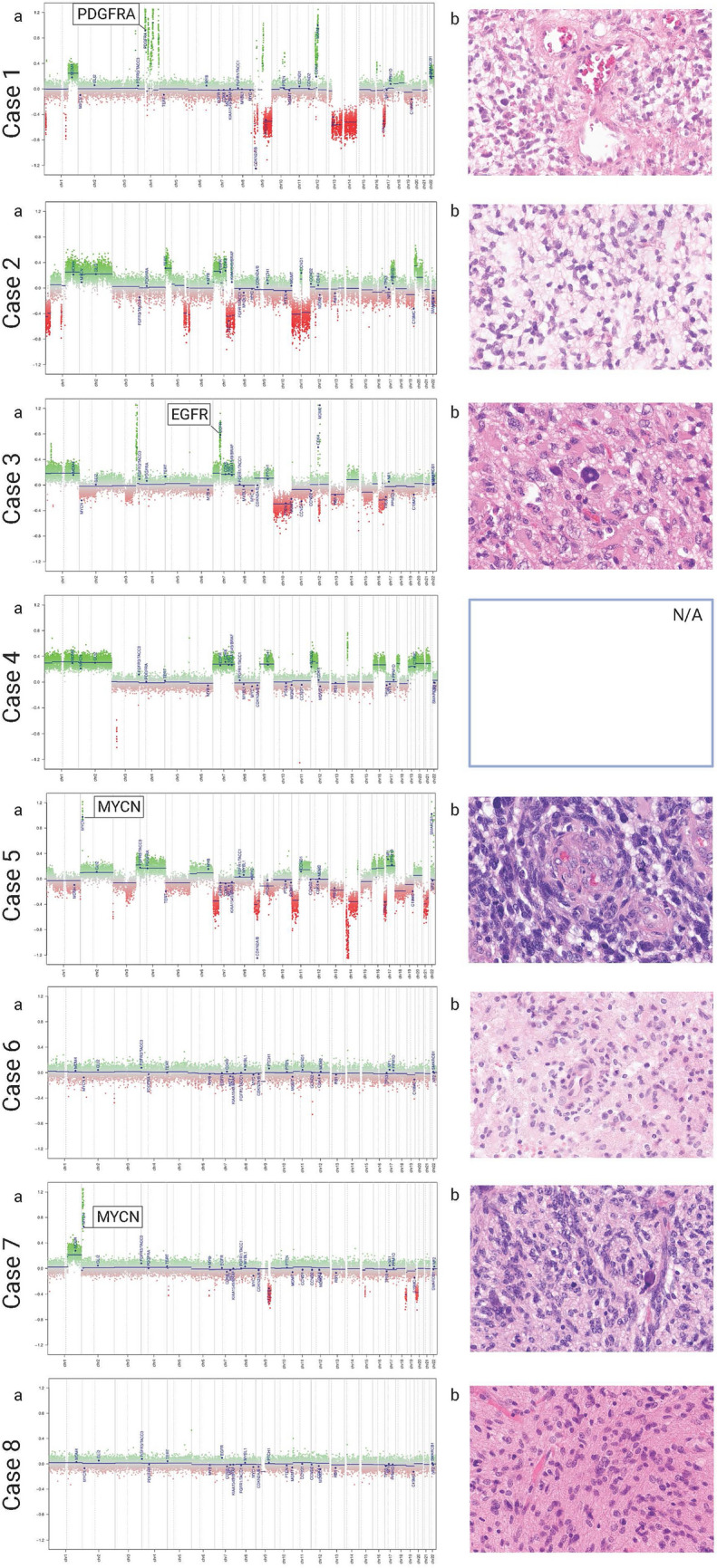
Hematoxylin–eosin stainings and copy number plots of the eight tumor tissues. *EGFR* epidermal growth factor receptor, *PDGFRA* platelet-derived growth factor receptor alpha

Figure 2 shows the hematoxylin–eosin stainings and copy number plots of the eight tumors.

**Fig. 3:**
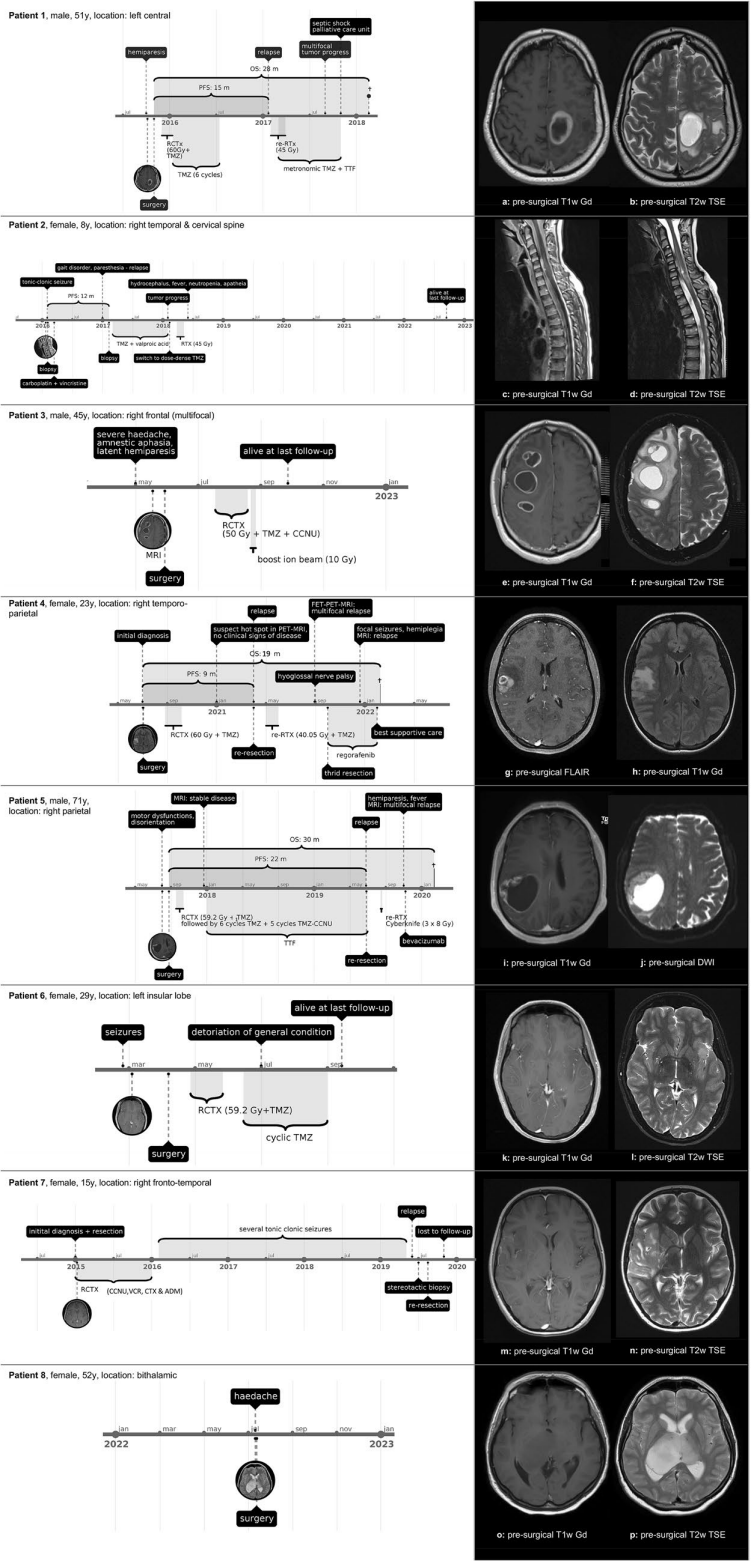
Illustration of patient’s clinical course, including preoperative MR-imaging. *ADM* adriamycin, *CCN*U lomustine, *CTX* cyclophosphamide, *DWI* diffusion-weighted imaging, *Gd* gadolinium, *Gy* gray, *MRI* magnetic resonance imaging, *n* native, *OS* overall survival, *PFS* progression-free survival, *RCTX* radiochemotherapy, *RTX* radiotherapy, *T1w* T1 weighted, *T2w* T2 weighted, *TMZ* temozolomide, *VCR* vincristine, *y* years

Figure 3 illustrates each patient’s clinical course, including preoperative radiologic imaging.

#### Imaging characteristics

Table 2 summarizes preoperative imaging features of all cases: all lesions appear hyperintense on T2 weighted (T2w) MRI imaging. Diffusion-weighted imaging (DWI) shows no diffusion restriction in any case. Other imaging phenomenology in pHGG is inconsistent. Some cases exhibit rim enhancement (patients 1, 3, 4, 5), others do not. Three out of eight show no enhancement at all (patients 6, 7, 8). Lesions present hypo- to isointense on native T1-weighted (T1w) MRI. In five cases tumor margins are sharp (patients 1, 3, 4, 5, 8) whereas three patients have a diffusely infiltrating tumor (three patients 2, 6, 7). Perilesional edema is present in seven cases (patients 1, 2, 3, 4, 5, 6, 7). Three cases demonstrate a cystic tumor (patients 1, 3, 5), all other cases display no signs of cysts or necrosis.

#### Patient 1

In October 2015, a 51-year-old male presented with right-sided hemiparesis. Cerebral MRI showed a left central necrotic lesion with perifocal edema (see Fig. 3A, B). Neuropathological examination confirmed the diagnosis of a glioblastoma multiforme, MGMT-promoter not methylated. The tumor was later identified as diffuse paediatric-type high-grade glioma, H3-wildtype and IDH-wildtype (pHGG RTK1; 12.5 classifier score 0.70). The patient received adjuvant radiochemotherapy according to the Stupp protocol [5] until July 2016. At the end of January 2017, imaging revealed a multifocal relapse. The patient underwent a second course of radiotherapy (single doses of 1.8 Gy up to a total dose of 45 Gy), metronomic temozolomide (20 mg/m^2^ b.i.d.) and tumor-treating fields (TTF). Six months later, in September 2017, MRI again showed multifocal tumor progression. The patient wished to continue metronomic temozolomide and TTF. Two months later his general condition deteriorated. He was taken to the intensive care unit due to a septic shock caused by an erysipelas of the right lower leg. The patient died in a hospice, 28 months after the initial diagnosis.

#### Patient 2

In February 2016 an 8-year-old girl was externally diagnosed based on histomorphology with a disseminated leptomeningeal oligodendroglial tumor after a tonic–clonic seizure. The patient received carboplatin- and vincristine-based chemotherapy. Almost one year later, in January 2017, she experienced a progressive gait disorder and paresthesia in the right hand. MRI demonstrated local tumor progression as well as intramedullary spinal metastases (see Fig. 3C, D). Neuropathological examination after a stereotactic biopsy now showed an anaplastic astrocytoma, IDH-wildtype with molecular features of glioblastoma, IDH-wildtype. DNA-methylation analysis was performed and the tumor was re-classified as glioblastoma, IDH-wildtype, subclass midline (brain tumor classifier v11b4 Score 0.75). Later, in the 12.5 version of the brain tumor classifier the tumor was re-classified as diffuse paediatric-type high-grade glioma, H3-wildtype and IDH-wildtype (pHGG, RTK1; 12.5 classifier score 1.0).

Postoperatively, the patient suffered from high intracranial pressure and the insertion of an external ventricle drain was performed. After prolonged convalescence, the girl was able to return to school but reported mild difficulties with writing and concentration. Dose-dense temozolomide (50 mg/m^2^/day, 21-days on/7-days off) and valproic acid (30 mg/kg/day) were administered from March 2017 to February 2018, when imaging again showed intracranial tumor progression. Chemotherapy was changed to temozolomide (140 mg/day, 5 days on/23 days off) and radiotherapy was administered (total dose of 45 Gy in single doses of 1.8 Gy). In June 2018, the girl presented with progredient hydrocephalus, fever, and neutropenia. Treatment included shunt revision and antibiotics. The patient seemed gradually more apathetic, even though imaging showed no signs of progression. Soon temozolomide had to be paused due to myelotoxicity. The patient was alive at last follow-up, 79 months after the initial diagnosis.

#### Patient 3

In May 2022 a 45-year-old male was admitted to our emergency department due to a sudden onset of severe headache, amnestic aphasia and a latent hemiparesis. MRI showed two multicystic, post-contrast rim-enhancing lesions in the right frontal lobe with extensive perifocal edema and signs of increased intracranial pressure (see Fig. 3E, F). The two lesions were microsurgically resected. Neuropathological examination revealed a glioblastoma, IDH-wildtype, MGMT-promoter methylated. DNA-methylation analysis was performed and the tumor was re-classified as glioblastoma, IDH-wildtype (brain tumor classifier v11b4 score 0.99). Later, in the 12.5 version of the brain tumor classifier, the tumor reached a classifier score of 0.89 for a diffuse paediatric-type high-grade glioma, H3-wildtype and IDH-wildtype (pHGG RTK2). The patient underwent radiochemotherapy with single fractions of 2 Gy up to a total dose of 50 Gy, followed by a 10 Gy heavy ion boost in 2 Gy single fractions and concomitant temozolomide and lomustine (CCNU) [6]. During the fourth cycle of adjuvant chemotherapy in November 2022, the patient developed epileptic seizures. He was alive at the last follow-up, five months after surgery without imaging signs of tumor progression.

#### Patient 4

In January 2021, a 22-year-old female patient presented herself at our institution. After a diagnosis of a right-parietal glioblastoma, IDH-wildtype in July 2020 (see Fig. 3G, H), she had undergone concomitant radiochemotherapy according to the Stupp regimen [5]. Presentation at our institution was made during the third cycle of adjuvant temozolomide. The patient had no clinical signs of disease, but fluoroethyl-l-tyrosine (^18^F)*-*positron emission tomography (FET-PET)-MRI showed an area of increased tracer uptake in the resection cavity. We agreed on the observation of the suspicious lesion and recommended the continuation of temozolomide. Four months later, in April 2021, the patient underwent re-resection of multifocal tumor relapse. Neuropathological analysis confirmed the diagnosis of glioblastoma, IDH-wildtype, MGMT-promoter not methylated. Because of a nuclear loss of PMS2 staining there was the suspicion of a diffuse paediatric-type high-grade glioma, H3-wildtype and IDH-wildtype [1]. DNA-methylation analysis was performed and the tumor was classified as diffuse paediatric-type high-grade glioma, H3-wildtype and IDH-wildtype in the 12.5 version of the Brain Tumor Classifier (pHGG MYCN, 12.5 classifier score 0.91). A second radiochemotherapy (median total dose of 40.05 Gy with single doses of 2.67 Gy) with metronomic temozolomide (20 mg/m^2^ b.i.d.) was initiated.

Four months later, the patient presented with a right hypoglossal nerve palsy. FET-PET-MRI revealed a multifocal tumor progression. Re-resection was performed in October 2021, which was well tolerated and adjuvant regorafenib was initiated [7]. After the first cycle, the patient presented with intermittent focal seizures, spastic hemiplegia on the left side and Cushing syndrome. MRI showed tumor progression. In February 2022, the patient was transferred to the palliative care unit, regorafenib treatment was terminated and best supportive care was initiated. The patient died 19 months after the initial diagnosis.

#### Patient 5

In August 2017, a 71-year-old male patient presented with sudden onset motoric dysfunctions and disorientation. MRI showed a large right temporoparietal hemorrhagic, cystic lesion (see Fig. 3I, J). The tumor was subtotally resected. Post-surgical MRI exhibited residual disease. Neuropathological examination revealed a glioblastoma, IDH-wildtype, MGMT-promotor methylated. The tumor profile was not classifiable in the 11b4 version of the brain tumor classifier. In the 12.5 version, the profile was classified as diffuse paediatric-type high-grade glioma, H3-wildtype and IDH-wildtype (pHGG RTK1; 12.5 classifier score 0.90) We initiated radiochemotherapy in an accelerated hyperfractionated scheme with single doses of 1.6 Gy b.i.d. to a total dose of 59.2 Gy and concomitant temozolomide, followed by one cycle of temozolomide, five cycles of combined temozolomide/CCNU and TTF [6]. Twenty-two months later, in June 2019, MRI showed tumor relapse in the right frontal and pa*****rietal lobe. Re-resection and adjuvant hypofractionated therapy via robotic radiosurgery (3 × 8 Gy, prescribed to the 70% isodose) was performed. Metronomic temozolomide (20 mg/m^2^ b.i.d.) was prescribed after radiotherapy. Five months later, in November 2019, the patient was admitted to our emergency department with left-sided hemiparesis, fever, elevated infection parameters and general malaise. MRI revealed massive tumor progression. Antibiotic treatment of the infection led to rapid reconstitution. The patient was released into outpatient care and died in February 2020, shortly after the initiation of bevacizumab treatment, 30 months after the initial diagnosis.

#### Patient 6

In February 2022, a 29-year-old female patient was admitted with new onset of high frequent seizures. MRI showed a left insular lesion, interpreted as focal cortical dysplasia (see Fig. 3K, L). FET-PET displayed an increased tracer-uptake suggestive of a high-grade glioma. Two months later, in April 2022, the patient underwent microsurgical resection. DNA-methylation analysis revealed glioblastoma, IDH-wildtype (v11b4 classifier score 0.93) and later, in the 12.5 classifier version, a diffuse paediatric-type high-grade glioma, H3-wildtype and IDH-wildtype (pHGG RTK2; 12.5 classifier score 1.0). Next-generation sequencing using the Illumina TruSight Oncology (TSO500) Panel showed five pathogenic mutations and one potential pathogenic mutation (CBL c.1096-1G > T [NM_005188.3]; NF1 c.372T > A; p.C124 [NM_001042492.2]; ANKRD11 c.4300G > T; p.E1434 [NM_013275.5]; BCOR c.3621dup; p.Q1208fs, [NM_001123385.1]; TERT c.G228A; NF1 c.5534T > G; p.I1845S [NM_001042492.2]). Adjuvant therapy comprised radiochemotherapy in an accelerated hyperfractionated schema with single doses of 1.6 Gy b.i.d. up to a total dose of 59.2 Gy and concomitant temozolomide, followed by a cyclic regimen according to Stupp et al. [5].

After initiation of the cyclic temozolomide treatment in July 2022, the patients' overall condition started to deteriorate when she developed pancytopenia and a severe SARS-CoV2-infection. Seizure medication had to be escalated and temozolomide had to be paused after the second cycle due to severe nausea, vertigo and headaches. MRI from November 2022, however, showed no tumor progression. The patient was alive at last follow-up, 6 months after surgery.

#### Patient 7

In August 2019, a 15-year-old female patient was referred to our neurosurgery department due to tumor relapse of a right fronto-temporal lesion (see Fig. 3M, N) which had been primarily resected abroad in 2015. At the time, external histological examination suspected a spindle cell carcinoma or gliosarcoma. Until 2016, the patient had received radiochemotherapy comprising CCNU, vincristine, cyclophosphamide and adriamycin. For the next two years, she experienced several tonic–clonic seizures. In June 2019, MRI showed a tumor relapse. External stereotactic biopsy was interpreted as spindle-cell astrocytic glioma with desmoplasia. In August 2019 we performed microsurgical re-resection. DNA-methylation analysis revealed a glioblastoma, IDH-wildtype, subclass MYCN glioblastoma (v11b4 classifier score 0.99, subclass score 0.98). The MGMT-promoter was not methylated. The case later scored 1.0 for the methylation class diffuse paediatric-type high-grade glioma, H3-wildtype and IDH-wildtype (pHGG MYCN) in the 12.5 classifier version. Adjuvant therapy was administered abroad and the patient was lost to follow-up.

#### Patient 8

A 52-year-old female patient was admitted to our hospital with the chief complaint of severe headache. MRI demonstrated a T1 hypointense and FLAIR hyperintense bithalamic lesion (right > left) with no contrast-enhancement (see Fig. 3O, P). In July 2022, a stereotactic biopsy was performed. DNA-methylation analysis showed a diffuse paediatric-type high-grade glioma, H3-wildtype and IDH-wildtype, subtype A&B (v12.5 classifier score 0.96). Immunohistochemical stains with antibodies against H3 K27M and H3 p.27me3 were performed during routine diagnostics. H3 K27M mutation was not present and H3 trimethylation was retained. A TERT promoter C228T mutation was detected by Sanger sequencing. Next-generation sequencing using the Illumina TruSight Oncology (TSO500) Panel revealed two additional, likely pathogenic mutations (BCOR c.4050C > A; p.Y1350 [NM_001123385.1]; CREBBP c.4337G > A; p.R1446H [NM_004380.2]) and an EGFR exon 18–25 kinase domain duplication (EGFR-KDD). Interdisciplinary tumor board recommended radiochemotherapy and TTF. Further treatment took place at an external hospital where it was agreed to postpone radiochemotherapy and try a watch and scan approach as the patient had no clinical signs of disease (Karnofsky performance score (KPS) 100%). MRI from November 2022 showed no tumor progression. The patient was alive at the last follow-up, four months after the initial diagnosis.

## Discussion

In 2015, Korshunov et al. presented a molecular analysis of 202 paediatric glioblastomas to investigate the prognostic significance of genomic and epigenetic alterations. The authors found a subset of 59 H3/IDH-wildtype pHGG patients with a median age of 12 years (range 1–18 years) [8].

Two years later, in 2017 the authors identified three distinct molecular subtypes, which differed in genomic and epigenetic profiles as well as in outcome. Although all analyzed tumors showed a dismal prognosis, the authors pointed out significant differences in survival among subtypes. So-called pHGG RTK2 tumors displayed the most favorable median OS of 44 months, followed by pHGG RTK1 with an intermediate median OS of 21 months and pHGG MYCN with the shortest median OS of 14 months [3].

In the same year Mackay and colleagues conducted a molecular meta-analysis of 1067 cases of pHGG and diffuse intrinsic pontine glioma. The median age of the population was 9.8 years, 982 were 21 years old or younger. They found that H3/IDH1-wildtype tumors had a 2-year survival of 23.5% and a median OS of 17.2 months. The authors recommended further investigation of the heterogenous subgroups [2].

Our patient group was significantly older than the published cases thus far and our oldest patient was diagnosed at age 71. As already mentioned in the WHO pHGG article, the prevalence in adults and elder patients might be underestimated [1].

Information on pHGGs radiological appearance is scarce. The WHO describes pHGGs MRI characteristics resembling other high-grade gliomas, showing contrast-enhancing tumors with mass effect [1]. So far, only the aspect of pHGG subtype MYCN has been specifically described in the literature [9, 10]. In 2019, Tauziède-Espariat et al. presented six cases of high-grade glioma (HGG)-MYCN with a median age of 3.7 years (range 1–7 years). The tumors were localized in the pons, mesencephalon and the middle cerebellar peduncle. On MRI HGG-MYCN exhibited signs of necrosis, rim-enhancement after contrast application and diffusion restriction. Median OS was 4.2 months [10]. In 2020, the authors examined five paediatric supratentorial HGG-MYCN cases and assembled them with 59 pHGG-MYCN cases in the literature. They found that pHGG MYCN were well circumscribed, showing only a small perilesional edema and homogenous contrast-enhancement. The tumors were described as solid and hypercellular with a diffusion restriction in the main part. Median PFS for pHGG-MYCN was reported to be 9 months, median OS was 16.6 months in this group. The authors suggested adding MYCN and IDH2 analysis to the standard molecular diagnosis of H3/IDH-wildtype malignant supratentorial tumors via DNA methylation profiling [4].

The cases in our series seemed to be universal in appearance, showing hypo- to iso-intensity in T1w, hyperintensity in T2w images and differing contrast agent enhancement behavior. In conjunction with the small case number, it is difficult to work out general MR imaging patterns; MR morphological findings are rather unspecific and not helpful in confirming or refuting a diagnosis.

Patients with suspected pHGG should receive a standard CNS tumor assessment protocol including T2w and T1w sequences e.g., TSE or MPRAGE, FLAIR or TIRM, DWI, and postcontrast T1w sequences e.g., TSE or MPRAGE. Additional PET might improve the specificity of diagnosis in cases where no certain tumor signs are seen on MR.

In 2020, Varlet et al. conducted a histopathological data analysis of 178 high-grade glioma patients (aged 3–18 years) and found that WHO grade 3 versus 4, including endothelial proliferation and necrosis, had no prognostic value in pHGG. However, a high Ki-67 index (≥ 20%) and midline location were significantly associated with dismal outcome [11].

As brain tumors are increasingly defined by their molecular profile, future subtype classification and evaluation of proliferation markers of pHGG might help to further refine treatment and determine prognosis.

In our study group, three patients died from their disease, with an overall survival of 19, 28 and 30 months after initial diagnosis. Four patients were alive at last follow-up, 4, 5, 6 and 79 months after surgery. One patient was lost to follow-up. PFS was 9, 12, 15 and 22 months. Until last follow-up, three patients remained without radiological tumor progress 4, 5 and 6 months after initial surgery.

Our data of eight diffuse paediatric-type high-grade glioma cases suggest that this entity is not confined to children and adolescents. Imaging showed no specific features. The diagnostic pathway is constrained to DNA methylation profiling. By reporting detailed procedural information, we might contribute to clinical decision-making until larger case series are published.

## Data Availability

Data are available on request from the authors.

## Abbreviations

CCNU: Lomustine
CNS: Central nervous system
CTX: Cyclophosphamide
DWI: Diffusion-weighted imaging
EGFR: Epidermal growth factor receptor
FET-PET: Fluoroethyl-l-tyrosine (^18^F)*-*positron emission tomography
FLAIR: Fluid attenuated inversion recovery
Gy: Gray
H3: Histone H3
HGG: High-grade glioma
IDH: Isocitrate dehydrogenase
KI-67: Antigen KI-67
KPS: Karnfosky performance status
MGMT: O^6^-methylguanine-DNA-methyltransferase
MPRAGE: Magnetization prepared rapid gradient echo
MRI: Magnetic resonance imaging
NGS: Next-generation sequencing
OS: Overall survival
PC: Paclitaxel and carboplatin
PDGFRA: Platelet-derived growth factor receptor A
PFS: Progression-free survival
PHGG: Diffuse paediatric-type high-grade glioma
T1w: T1-weighted image
T2w: T2-weighted image
TIRM: Turbo inversion recovery magnitude
T-SNE: T-distributed stochastic neighbor embedding
TTF: Tumor-treating fields
WHO: World Health Organization
